# Fluorescent-Dye-Labeled Amino Acids for Real-Time Imaging in *Arabidopsis thaliana*

**DOI:** 10.3390/molecules28073126

**Published:** 2023-03-31

**Authors:** Yao Yuan, Fuxiang Cao, Guangming Yuan

**Affiliations:** 1 College of Science, Central South University of Forestry and Technology, Changsha 410004, China; yaoyuancsu@msn.cn (Y.Y.);; 2 College of Horticulture, Hunan Agricultural University, Changsha 410128, China

**Keywords:** *Arabidopsis thaliana*, fluorescent imaging, dye-labeled, amino acid uptake

## Abstract

Amino acid is the main transport form of reduced nitrogen in plants. To investigate the uptake and source–sink translocation process of plants to help understand their physiological roles and transport mechanisms, we designed and synthesized three fluorescent-dye-labeled amino acids as tools to visualize amino acid transportation in *Arabidopsis thaliana*; these amino acids consist of amino acids linked to the fluorophore nitrobenzoxadiazole (NBD) with excellent optical properties. Furthermore, we incubated *Arabidopsis thaliana* with these NBD fluorescent-dye-labeled amino acids for real-time imaging along with fluorescence enhancement for 24 h. The results showed that *Arabidopsis thaliana* could absorb them directly from the roots to the leaves. Therefore, our fluorescent-dye-labeled amino acids provide a de novo tool and strategy for visualizing amino acid absorption and transportation in plants.

## 1. Introduction

Fluorescence imaging technology has been widely used in biological imaging research, due to its advantages of high specificity, quick image, deep tissue penetration, and so on. With fluorescence imaging technology, target molecules can be observed and analyzed without changing the physiological state of the sample, so it can be used to study continuous life processes. The binding of fluorescent groups to biomolecules enables their subcellular and tissue-specific localization, as well as the sensitive and specific visualization of dynamic changes in plants, and therefore represents a powerful tool for plant physiological research. Fluorescence imaging in plants is used for the study of a wide range of physiological features, such as plant hormones [1], gene expressions [2], stress responses [3,4], and membrane microdomains [5]. There are reports on the absorption and tracking of elements in plant transport systems. There are reports in which four NBD fluorescent-dye-labeled auxins were synthesized and found to show dose-dependent inhibition of auxin-induced effects [1]. In the fluorescent images, these analogs exhibited a tissue auxin gradient in the roots. Pollen CDs synthesized via a hydrothermal method were used for monitoring nitrogen, phosphorus, and potassium elements by tracking them in *Brassica parachinensis* L. via fluorescent imaging and transmission electron microscopy [6]. Carbon dots marked the biological transport systems and enriched the periplasmic space, significantly increasing photosynthesis efficiency and soluble sugar content. However, the application of fluorescence imaging technology in plants is often complicated by the strong endogenous autofluorescence emission of plant tissues along with the impermeability of plant cell membranes and walls to protein-based labels and macromolecules. These obstacles limit the application of fluorescence imaging technology in plant species. Therefore, it is necessary to optimize plant imaging techniques with better accuracy, selectivity, biocompatibility, and spatiotemporal resolution.

**Figure 1:**
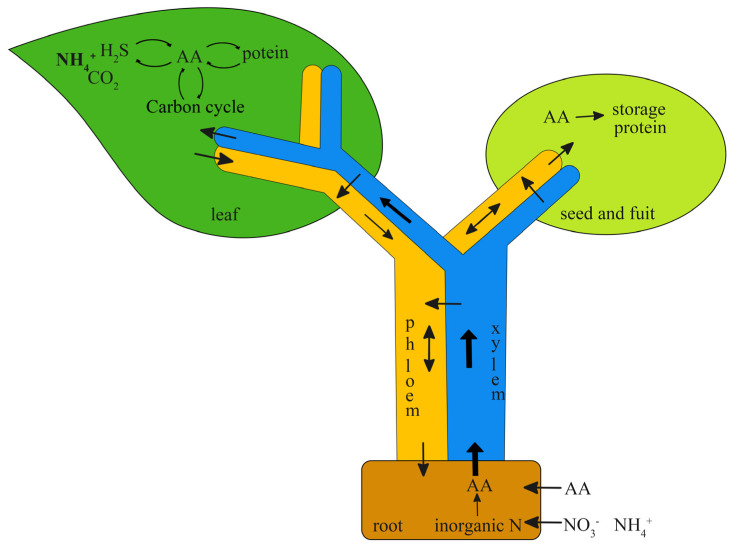
Schematic overview of the key steps in uptake and translocation of amino acids (AAs) in *Arabidopsis*.

Plants take up both inorganic nitrogen (e.g., ammonium and nitrate) and organic nitrogen (e.g., amino acid and small polypeptide) from soil [7,8,9]. Organic nitrogen is transported in the form of amino acids in most plant species [9]. Amino acids can be taken as direct N sources for plants, but our knowledge of the nutrition machinery in organic nitrogen is limited compared with inorganic nitrogen. Recent studies have demonstrated that altering amino acid allocation processes impacts N and C assimilation, plant growth, and seed development [10,11]. Due to the role of transporters in long-distance transportation through roots, leaves, and seeds, these genes may be good modification targets for crop improvement. Figure 1 shows the schematic uptake, translocation, and metabolism process in plants. The uptake of amino acids in roots from the soil is powered by the proton gradient on the plasma membrane and facilitated by amino acid transporters [12], and they are mainly transported via the xylem route to shoots along with transpiration flow. Along the long-distance transport pathway, the stream transfer from the xylem to the phloem and is then unloaded into leaves, flowers, seeds, and fruits. Since transpiration is highest in photosynthetically active leaves, the bulk flow of amino acids is allocated with the xylem transpiration stream to leaves. In leaves, amino acids are imported into the mesophyll cells, where they are utilized or stored as N nutrition storage. The exported amino acids from leaves are loaded into the phloem of minor veins for redistribution to other tissues. Meanwhile, amino acids are also synthesized in roots and leaves via N reduction, photorespiration, and protein hydrolysis. The intercellular and intracellular transmembrane pathways in the process of amino acid uptake and transport require the facilitation of transporter proteins on the plasma membrane. They play roles in the cellular introduction of amino acids that are transported together with protons. The expression of transporter proteins showed respective tissue localization specificity and substrate selectivity. Some active transporter proteins in the root have been determined in *Arabidopsis*, such as lysine and histidine transporters (*LHT1* and *LHT6* [8,13]), amino acid/auxin permease (*AAP1* and *AAP5* [8,14]), and proline transporter (*PROT2* [9]). They mediate the uptake of free amino acids in soil into the root.

The absorption and allocation of amino acids in plants are mainly studied using radioactively labeled methods, which depend on ^15^N and ^13^C isotope labeling. These methods can give specific experimental data and be used to calculate the kinetics of amino acid uptake and translocation in plants. Nevertheless, these methods should be operated under sterile conditions. Amino acids can be both absorbed by plants and utilized by microorganisms, and there is fierce competition. Radiolabeled amino acids can be decomposed into small fragments containing ^15^N and ^13^C by contaminated microorganisms during a long culture period. Both amino acids and small fragments can be taken up by plant roots. Furthermore, post-uptake plant metabolism also affects the isotope-labeled quantification [15]. The rationality of these radioactively labeled methods is still disputed [15,16].

Until now, there has been no fluorescent label used to track amino acids to study the uptake, transport, and metabolism mechanism in plants. In this work, three fluorescent-dye-labeled amino acids were designed and synthesized as de novo tools for visualizing amino acids in *Arabidopsis thaliana*. Specifically, the fluorescent dye NBD-Cl with excellent optical properties reacted with three amino acids under mild conditions to produce fluorescent amino acids with strong fluorescence signals. The synthesis of these fluorescent amino acids is simple and has low costs and a high yield. The optical signal is stable and suitable for imaging in vivo. The fluorescent amino acids could be directly absorbed by *Arabidopsis thaliana* into the vascular bundles from roots to leaves and showed accumulation in shoots via real-time fluorescence imaging for 24 h. Thus, our fluorescent-dye-labeled amino acids can provide a new tool and strategy for visualizing amino acid absorption and transportation in plants.

## 2. Results and Discussion

### 2.1. Design and Synthesis of Fluorescent-Dye-Labeled Amino Acid Derivatives

The current understanding of the mechanism of substrate recognition characteristics of transporters is still obscure. The substrate specificity of plant amino acid transporters has been found to be attributed to amino groups and carboxyl groups. Generally, transporters show substrate diversity between and within transporter families. Some members of the *LAT* (L-type amino acid transporter) family have been identified to be transporters of polyamine and paraquat [17]. Glycinergic–fipronil was reported to be taken up across membranes mediated by amino acid importers and competitively suppressed by amino acids. The expressions of four amino acid transporter genes were induced by glycinergic–fipronil and suggested to be probably involved in its uptake [18]. These compounds are structurally different from amino acids despite the amine groups. To observe fluorescent signals in plants in vivo, fluorescent-dye-labeled amino acids should be designed to retain the stereo configuration of the amino acid segments and be as small as possible.

We first selected the most sensitive and small-size fluorescent dyes that maintain as high biocompatibility as possible. Previous studies of NBD-labeled auxin analogs showed the advantages of sensitive detection, good biological image resolution, and deep penetration in *Arabidopsis thaliana* with specialized localization [1]. The synthesis route of NBD with amino groups is simple in mild conditions at room temperature (RT) [19]. Since C- and N-terminal domains are often important interactive sites, the incorporation of the fluorescent label at a well-chosen site is critical to avoid conformational and biological characteristic changes. Glycine, lysine, and proline were chosen because they are neutral amino acids that are high and moderate affinity substrates of *LHT1*, *LHT6*, and *AAP1*, which are currently known major transporters in root function for amino acid uptake. They have simple substituents, fewer side reactions, and result a higher yield with NBD-Cl.

**Figure 2:**
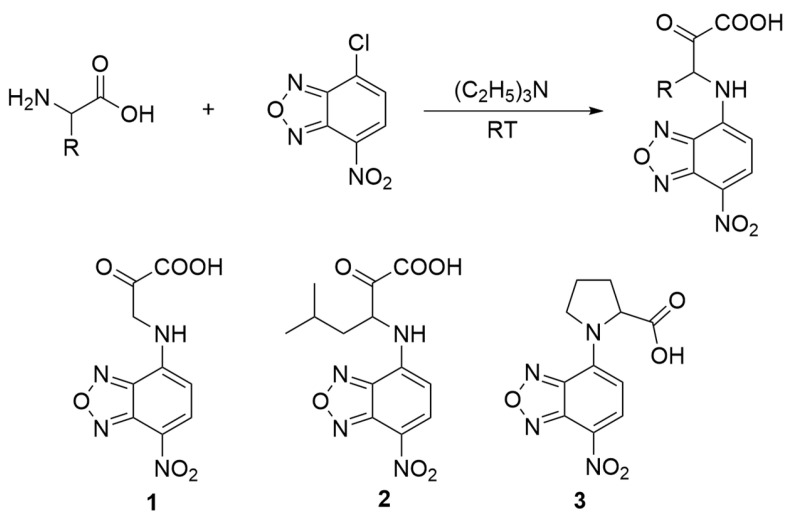
Synthesis route of fluorescent-dye-labeled amino acid derivatives **1**–**3**.

NBD-Cl reacts well with amino acids under mild conditions, with yields of 20–40%. Three fluorescent-dye-labeled amino acids were synthesized using the procedure shown in Figure 2. The C–N bonding at the N-terminal prevents amino acid breakdown due to the natural amino group degradation, enabling us to visualize the sensitivity of fluorescent-dye-labeled amino acids via simple manipulation. All compounds were characterized using ^1^H NMR and ESI-MS (see Supplementary Materials).

### 2.2. Interference and Effect of pH Value on Fluorescent-Dye-Labeled Amino Acid Derivatives

**Figure 3:**
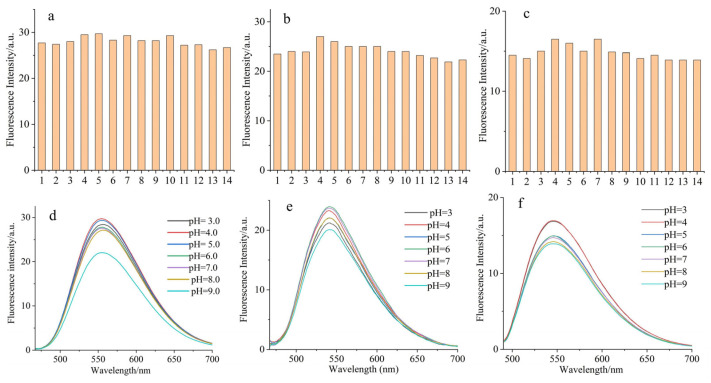
(**a**–**c**) Changes in fluorescence emission spectra of 2 μM NBD-labeled amino acid in 20 μM ions and biomolecules PBS/EtOH solutions. The numbers from 1 to 14 correspond to untreated samples, NaCl, KNO_3_, CaCl_2_, MgSO_4_, FeCl_3_, CuSO_4_, Vc, H_2_O_2_, ZnAC, glucose, GSH, Cys, and Hcys; (**d**–**f**) changes in fluorescence emission spectra of 2 μM NBD-labeled amino acid in 10 mM PBS different pH solutions. Respective excitation wavelengths of each derivative: λ_ex1_ = 458 nm, λ_ex2_ = 458 nm, and λ_ex3_ = 481 nm.

Plant internal milieu comprises a variety of components, and molecules in cells undergo complex and highly dynamic chemical reactions and interactions. Anti-interference capability is important for application in vivo. Using the fluorescent-dye-labeled amino acids, a series of anions, cations, and biomolecules were tested for interference. As shown in Figure 3, the fluorescence intensity of these three fluorescent-dye-labeled amino acids showed hardly any interference after the addition of the analyte species. Therefore, the results demonstrated that these amino acid derivatives could meet the interference requirement in plants.

Furthermore, since the pH value in plants and soil fluctuates and distributes unevenly, the derivatives should have a suitable pH working range. We tested the effects of pH value on these derivatives in 10 mM PBS solutions. Approximate characteristic fluorescence could be observed in a pH value interval of 5.0–8.0, which is in the range of plant tissues’ pH variation [20]. These results imply that fluorescence of these derivatives would be stable despite pH value fluctuation in practical application. Thus, a partially neutral 10 mM PBS solution (pH = 7.0–7.4) was utilized in later tests.

### 2.3. Tissues and Whole-Plant Fluorescence Imaging

To demonstrate the application of this method in plants, staining was observed in the roots of *Arabidopsis thaliana* using a fluorescence microscope. Seedlings were watered with water solutions combined with fluorescent amino acid derivatives, and then the roots were observed using a fluorescence microscope. Roots emit autofluorescence derived from phenolics and lignins in cell walls.

**Figure 4:**
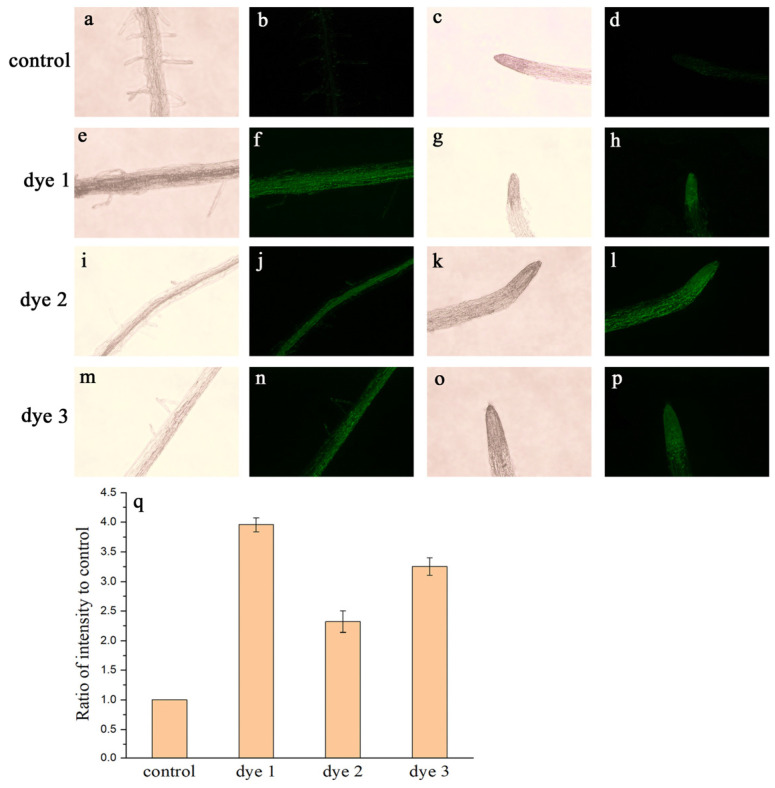
Microscope images of *Arabidopsis thaliana* roots treated with NBD fluorescent-dye-labeled amino acids: (**a**,**c**) bright-field images of untreated samples as control; (**b**,**d**) fluorescence channel images of untreated samples as control; (**e**,**g**) bright-field images of dye-1-treated roots; (**f**,**h**) fluorescence channel images of dye-1-treated roots; (**i**,**k**) bright-field images of dye-2-treated roots; (**j**,**l**) fluorescence channel images of dye-2-treated roots; (**m**,**o**) bright-field images of dye-3-treated roots; (**n**,**p**) fluorescence channel images of dye-3-treated roots; (**q**) quantification of the fluorescence emission intensity. Mean fluorescence emission intensity values were calculated from measurements of eight roots for each treatment.

Since the fluorescence strength reflects the concentration and density of fluorescent substances, an increase in the fluorescence strength could be attributed to the accumulation of fluorescent substances. Therefore, untreated roots were used as the control group. First, we observed the maturation zone coated by root hairs. The control group showed faint, uniform fluorescence in the root. All the treated roots (Figure 4f,j,n) exhibited markedly enhanced fluorescence in the green channel compared with the control group (Figure 4b). This implies that *Arabidopsis thaliana* can take up dye-labeled amino acids from the soil in an intact form. The staining showed some unique tissue selectivity, and the images showed concentrated fluorescence in the central column, which is the stele comprising the phloem and xylem. Meanwhile, there were faint signals in root hairs and moderate signals on the epidermis. A comparison of the partitioning of fluorescent amino acids to previous reports for amino acids indicates that the derivatives well reflect the enrichment of amino acids in vascular bundles [7,21,22]. Therefore, this method provides a visual representation of dye-labeled amino acid uptake from the soil as well as translocation via vascular bundle in plant roots.

Root hairs project from the root surface to increase the root’s superficial area to take up water and mineral nutrients from the soil. The uptake of nutrients in roots is mainly energized by the H^+^ gradient across the cell membrane, which is facilitated by H^+^-ATPase. It is obvious that hardly any fluorescent flows through root hairs. Then, we obtained fluorescent images of the root tip to observe absorption in other parts of the root. The fluorescence strength in the root tip increased dramatically. Figure 4h,l,p show the concentration gradients in the root tips. The exogenously applied fluorescent amino acids preferentially accumulated in the root tip area, indicating that the epidermis of the root tip is an active zone for absorption. The fluorescence aggregation area stretches over the root cap and meristematic zone, which are coated by multiple layers of parenchyma cells. According to the above results, our fluorescent amino acids can be absorbed by the root epidermis and shows a strong affinity to the parenchyma cells of the root tip. The root tip is an active zone of the root for nutrient absorption, synthesis, and secretion of organic nutrients. Xylem loading in the root was described to result in the export of amino acids from the endodermis, pericycle, and vascular parenchyma to the apoplast, which is generally via a passive transport pathway [22]. However, little is known about the absorption mechanism in the root, as well as their sensing and signaling mechanisms. Fluorescence imaging showed the absorption zone and aggregation mode of fluorescence-labeled amino acids in the root.

**Figure 5:**
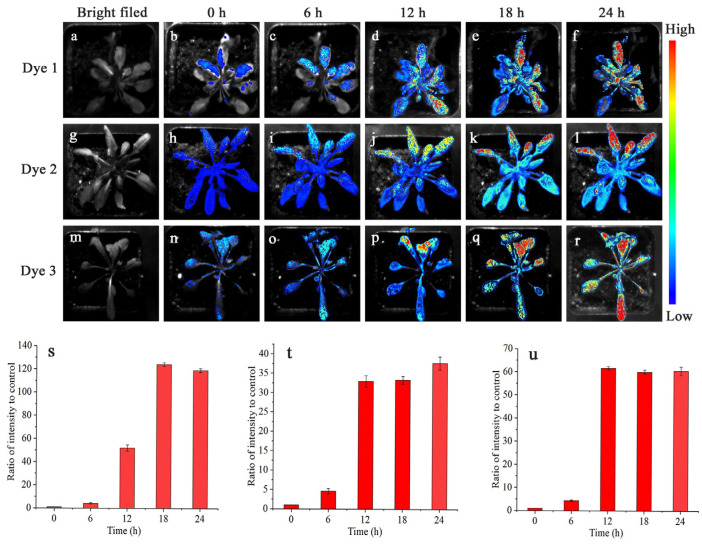
Whole-plant real-time imaging (pseudo-color) of *Arabidopsis thaliana* treated with 50 μM NBD amino acid–water solution: (**a**,**g**,**m**) bright-field images of plants; (**b**–**f**) real-time fluorescence channel images of dye-1-treated plants; (**h**–**l**) real-time fluorescence channel images of dye-2-treated plants; (**n**–**r**) real-time fluorescence channel images of dye-3-treated plants. Respective excitation wavelengths of each derivative: λ_ex1_ = 458 nm, λ_ex2_ = 536 nm, λ_ex3_ = 481 nm, and λ_em_ = 500–560 nm, scale bar: 10 μm; (**s**–**u**) quantification of the fluorescence emission intensity. Each treatment was repeated 3 times.

Plants have autofluorescence covering a wide spectral region; the blue and green fluorescence emissions come from phenolics covalently bound to cell walls, and red and far-red fluorescence emissions derive from chlorophyll in chloroplasts [23]. There are obstacles to applying fluorescence techniques in plants due to intense and wide-wave range autofluorescence. Therefore, in our experiment strategy, we assumed that fluorescent amino acids could be transported to the shoot through vascular bundles, where an increase in concentration would lead to an increase in fluorescent strength. We observed the accumulation of fluorescent amino acids by detecting the changes in fluorescence strength in the whole plant, which is reflected by color changes in the images. As shown in Figure 5, all plants emitted autofluorescence prior to treatment (0 h). A low concentration range does not allow for the visualization of fluorescence enhancement due to the strong autofluorescence. Thus, it is critical to choose a medium concentration for tests in vivo. Despite this shortcoming, the three fluorescent amino acids showed satisfactory performances at concentrations below 50 μM. This concentration covers a wide range of free amino acid concentrations in agricultural and forest ecosystems [14].

The fluorescence strength of all treatments increased in a time-dependent manner, directly demonstrating dye-labeled amino acid accumulation. In the early 18 h, the fluorescence strength rapidly and strongly increased and then slowed down in later hours. This is in agreement with previous reports on *Arabidopsis* and other plant species grown under different conditions. The rate of amino acid uptake via roots in individual species has a linear trend at the early stage, and the initial uptake rate is higher than the rate calculated with the later time points [16]. This is the first study to demonstrate root-derived amino acid uptake and partition procedure in living plants using the fluorescence technique. There were minor differences in the fluorescence enhancement rate related to the species of amino acids. There are some factors that affect amino acid uptake from the soil, such as amino acid species, concentrations of amino acid, and inorganic N nutrition [22,24].

NBD fluorescent-dye-labeled amino acid accumulation was initially detected in young leaves and thereafter in all leaves. In subsequent stages, the accumulation in old leaves was slower than that in some of the young leaves, indicating that young leaves are preferred for amino acid allocation. The amino acids absorbed from the soil or synthesized in roots are mainly transported via the vascular bundles to shoots along with transpiration flow. Since the transpiration rate is highest in photosynthetically active leaves, the bulk flow of amino acids from the root is allocated to the leaves. The absorption and allocation of amino acids require coordinated activities of transport proteins. A considerable number of amino acid transporters have been identified, and their expression differences and substrate selectivity in different organs have been described. Nutrient concentration and forms, as well as environmental stress, might induce N nutrition requirements and metabolism changes and then lead to the regulation of amino acid transporters [9,24,25,26]. In plant development, amino acids in photosynthetically active cells are upregulated to provide substrates with highly active protein synthesis. Efforts to study amino acid allocation patterns in specific cells and tissues help elucidate the potential signal network and metabolism responses to environmental stress. It remains unclear which metabolic pathways are involved in fluorescent amino acids. These efforts can also unveil new tools for amino acid metabolism analysis. Therefore, we will consider these issues in future research.

All these results show that fluorescently labeled amino acids perform well in the imaging of amino acid uptake and transport in *Arabidopsis thaliana*. Therefore, we could consider studying the metabolic mechanism with this strategy to reveal the role of the transport system in the distribution of organic nitrogen to specific tissues, cells, and compartments to clarify the potential signal network and provide strong evidence for the role of amino acids in plant growth, development, and productivity.

## 3. Materials and Methods

### 3.1. Reagents and Instruments

Reagents were purchased from Adamas (Basel, Switzerland), Acros (Pittsburgh, PA, USA), TCI (Tokyo, Japan), Sigma-Aldrich (St. Louis, MO, USA), and SCRC (Shanghai, China). All reagents were of analytical grade and obtained from commercial suppliers. Dichloromethane (CH_2_Cl_2_) was distilled over calcium hydride. Triethylamines were stored with molecular sieves (4A) before use. The water used in all experiments was purified using a Millipore Milli-Q water system (Billerica, MA, USA). ^1^H NMR (400 MHz) spectra were recorded on a Bruker Ascend 400 (Fällanden, Switzerland) in CDCl_3_ as a solvent with TMS as the internal standard. UV–vis absorption spectra were recorded on a Shimadzu UV-1800 spectrometer (Kyoto, Japan) in 1.0 cm path-length quartz. Fluorescence spectra were recorded on an Agilent Cary eclipse fluorometer (Santa Clara, CA, USA) with both excitation and emission slits set at 5 nm. The pH value measurements were carried out with a Mettler Toledo Delta 320 pH meter (Zurich, Switzerland). The fluorescence microscopy images of plant tissue were recorded with an Olympus CKX53 fluorescence microscope (Tokyo, Japan). Whole-plant real-time images were recorded with ImagingPhotonIMAGER (Paris, France). All experiments were performed in flame-dried glassware using dry solvents unless otherwise noted.

### 3.2. Synthesis of the Dye-Labeled Fluorescent Amino Acids

Under an argon atmosphere, the amino acids were treated with 1 equivalent of NBD-Cl and 2 equivalents of triethylamine as the base in dichloromethane at room temperature for 1 h. The reactions were monitored via thin-layer chromatography (TLC) using silica gel GF254, and the components were visualized using UV light (254 and 365 nm). The purification of the reaction mixtures was conducted via column chromatography with silica gel (100–200 mesh), eluted with a mixture of chloroform and methanol producing compounds **1**–**3** with 20–40% yield. Eluent composition is given for each substance. Powders were prepared via vaporization under reduced pressure. The compound structures were characterized using ^1^H NMR and MS spectrometry (see Supplementary Materials). The stock solutions of NBD amino acids in ethanol (C_2_H_5_OH) were stored in 5 mL microcentrifuge tubes wrapped with aluminum foil at −20 °C.

NBD–glycine (Dye 1): brown solid, 40% yield; ^1^H NMR (400 MHz, CDCl_3_): 8.03 (s, 1H); 6.41–6.39 (d, *J =* 8.0 Hz, 1H); 6.22–6.19 (d, *J =* 12.0 Hz, 1H); and 2.09 (s, 2H). ESI-(M + H)^+^: 239.3.

NBD–leucine (Dye 2): red solid, 34% yield; ^1^H NMR (400 MHz, CDCl_3_): 8.50–8.48 (d, *J* = 8.0 Hz, 1H); 6.41–6.39 (d, *J* = 8.0 Hz, 1H); 6.22–6.19 (d, *J* = 12.0 Hz, 1H); 4.88–4.85 (dd, *J* = 4.0 Hz, 2H); 3.50 (s, 1H); 2.26–2.23 (t, *J* = 6.0 Hz, 1H); and 1.07–0.99 (dd, *J*_1_ = 4.0 Hz, *J*_2_ = 8.0 Hz, 6H). ESI-(M + H)^+^: 295.2.

NBD–proline (Dye 3): dark brown solid, 20% yield; ^1^H NMR (400 MHz, CDCl_3_): 6.10–6.08 (d, *J* = 8.0 Hz, 1H); 5.39–5.37 (d, *J* = 8.0 Hz, 1H); 4.34–4.29 (m, 2H); 3.85 3.66 (m, 4H); and 3.45 (s, 1H). ESI-(M + H)^+^: 279.2.

### 3.3. Quantum Yield (QY) Estimation

Emission and absorbance spectra were acquired at a concentration of 2 μM in methanol. Fluorescence intensity was integrated into the region of 468–700 nm. The QY was calculated from the following formula [27]:

$$QY_{x}=\frac{F_{x}}{F_{s}}\frac{A_{s}}{A_{x}}\times \frac{n_{x}{}^{2}}{n_{s}^{2}}\times QY_{s}$$

where *F* and *A* refer to the integrated fluorescence intensity and absorbance, and *n* is the refractive index. The subscript s denotes the standard (2 μM rhodamine B in methanol).

### 3.4. Interference and Effect of pH Value on Fluorescent Amino Acids

Interference fluorescence spectra were measured in a 10 mM PBS/EtOH solution (99:1, *v*/*v*). The solutions of various testing species were prepared from NaCl, KNO_3_, CaCl_2_, MgSO_4_, FeCl_3_, CuSO_4_, Vc, H_2_O_2_, ZnAC, glucose, GSH, Cys, and Hcys, with PBS solutions at a concentration of 20 μM.

The effects of pH value were investigated in 10 mM PBS buffer solutions. The pH gradient buffer solutions from 3.0 to 9.0 were achieved by adding different volumes of 0.1 M HCl or NaOH solution. The fluorescence emission spectra of 2 μM NBD fluorescent-dye-labeled amino acids were recorded in different pH 10 mM PBS solutions.

### 3.5. Plant Material and Growth Conditions

Seeds of *Arabidopsis thaliana* ecotype Columbia-0 were grown in soil (vermiculite/high-quality soil = 1:3) under long-day light conditions (16 h light/8 h dark) at 20–22 °C and 40% relative humidity. To obtain seedlings, the seeds were germinated in wet vermiculite for 1–2 weeks.

### 3.6. Fluorescence Microscopy Imaging Investigations

For tissue fluorescence microscopy images, seeds were germinated in vermiculite. Briefly, 14-day-old seedlings were watered with the 3 treatments of 20 μM fluorescent amino acid–water solutions and incubated for 20 min. One group was treated with water as the control group. Finally, the whole plants were washed with 10 mM PBS three times and once with water. Fluorescence microscopy images of the roots were taken in the green channel. In order to investigate the absorption and transportation characteristics of the fluorescent-dye-labeled amino acids in *Arabidopsis thaliana* under natural conditions, the tests of plants were conducted considering 2–50 μM as naturally occurring concentrations [14].

### 3.7. Imaging Analysis of the Whole Plant

The plants were imaged after growing in soil for 6 weeks. The testing plants were watered with 50 μM fluorescent-dye-labeled amino acid–water solutions and grew in dark to avoid a breakdown in long-term exposure to light. The bright-field and fluorescence-field images were taken every 6 h for 24 h. The fluorescence-field images were obtained in the green channel.

## 4. Conclusions

We designed and synthesized three novel fluorescent-dye-labeled amino acid derivatives in the form of bonding with the fluorophore NBD. The emission spectra of these compounds were stable within the physiological interference range. We found that they were absorbed via the root into the vascular bundle and translocated to the whole-plant tissues along with the vascular system due to enhanced fluorescence strength. The fluorescence characteristics of these compounds can be used as a new strategy to track amino acid nutrient uptake and transportation through plants and advance our understanding of transport machinery. They may find applications in biotechnology, horticulture, agriculture, chemical fertilizer manufacture, etc.

## Data Availability

Not applicable.

## Supplementary Materials

The following supporting information can be downloaded at: https://www.mdpi.com/article/10.3390/molecules28073126/s1, Table S1: Quantum yield of fluorescent-dye-labeled amino acids; Figure S1: ^1^H-NMR spectrum o f compound **1**; Figure S2: ESI-MS spectrum of compound **1**; Figure S3: ^1^H-NMR spectrum of compound **2**; Figure S4: ESI-MS spectrum of compound **2**; Figure S5: ^1^H-NMR spectrum of compound **3**; Figure S6: ESI-MS spectrum of compound **3**.

## Sample Availability

Samples of the compounds are available from the authors.
