# Supplementary material for: Fluorescent-Dye-Labeled Amino Acids for Real-Time Imaging in Arabidopsis thaliana
Source: Molecules. 2023 Mar 31;28(7):3126. doi: 10.3390/molecules28073126 (PMC10095931; doi:10.3390/molecules28073126)
Supplement: Supplementary file 1 [file molecules-28-03126-s001.zip › molecules-2258153-supplementary.pdf]

Supplementary Materials: Fluorescent-Dye-Labeled Amino Acids for Real-Time Imaging in *Arabidopsis thaliana*

Yao Yuan, Fuxiang Cao and Guangming Yuan

Table S1. Quantum yield of fluorescent-dye-labeled amino acids

Compound	Quantum yield
1	0.6423
2	0.5783
3	0.4268

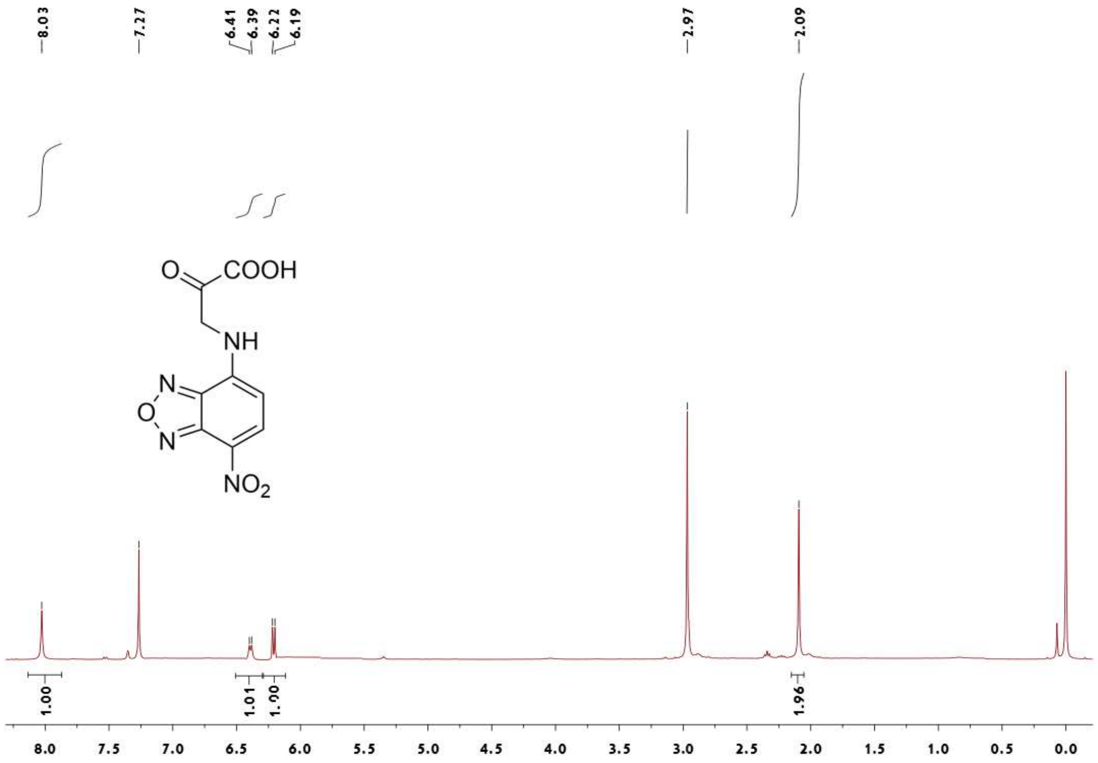

Figgure S1. ^{1}H-NMR spectrum of compound 1

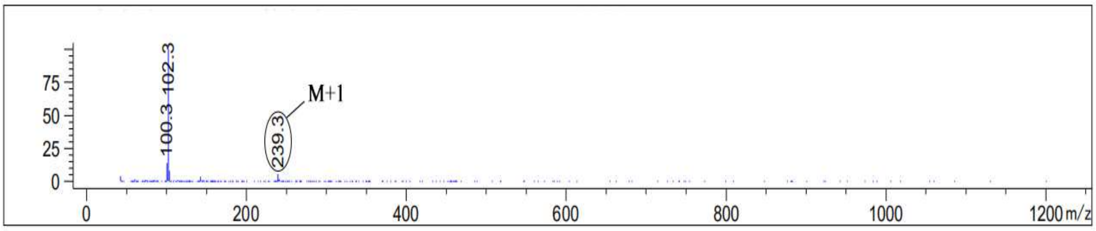

Figure S2. ESI-MS spectrum of compound 1

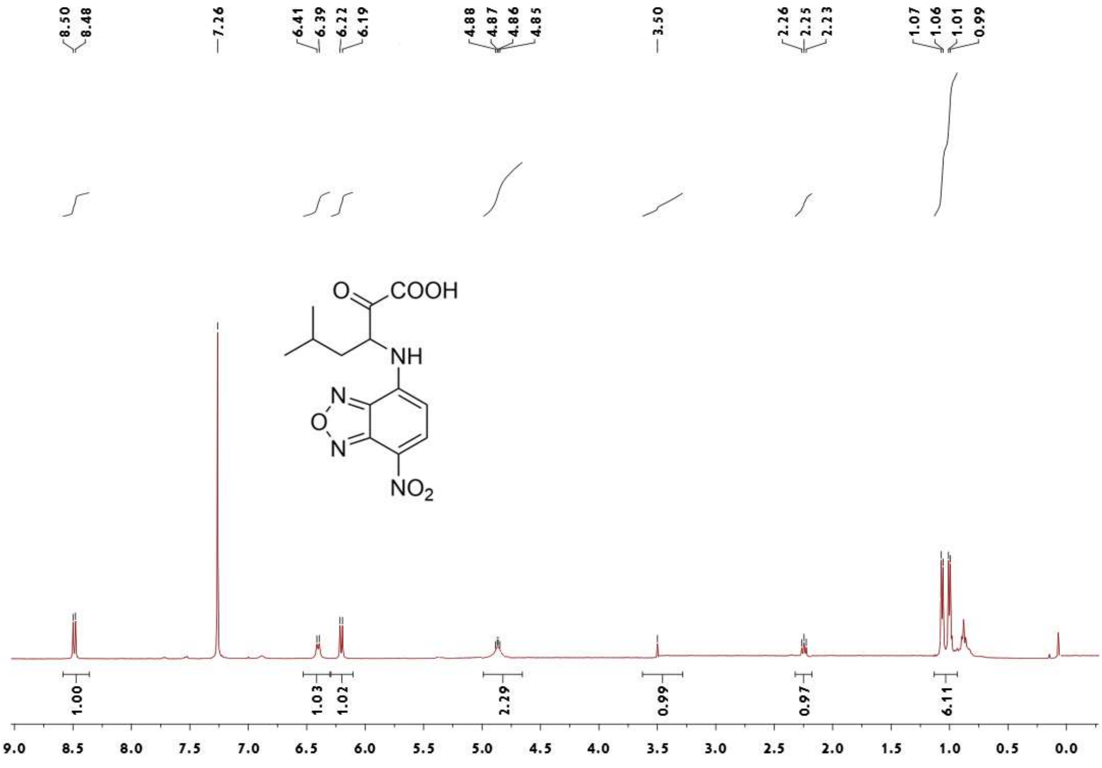

Figure S3. ^{1}H-NMR spectrum of compound 2

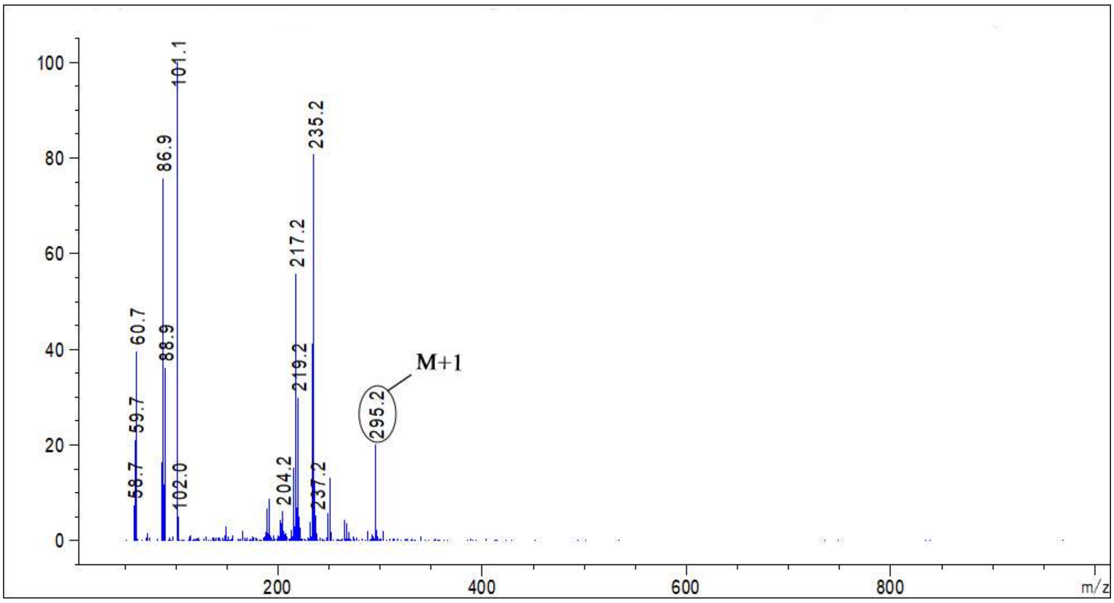

Figure S4. ESI-MS spectrum of compound 2

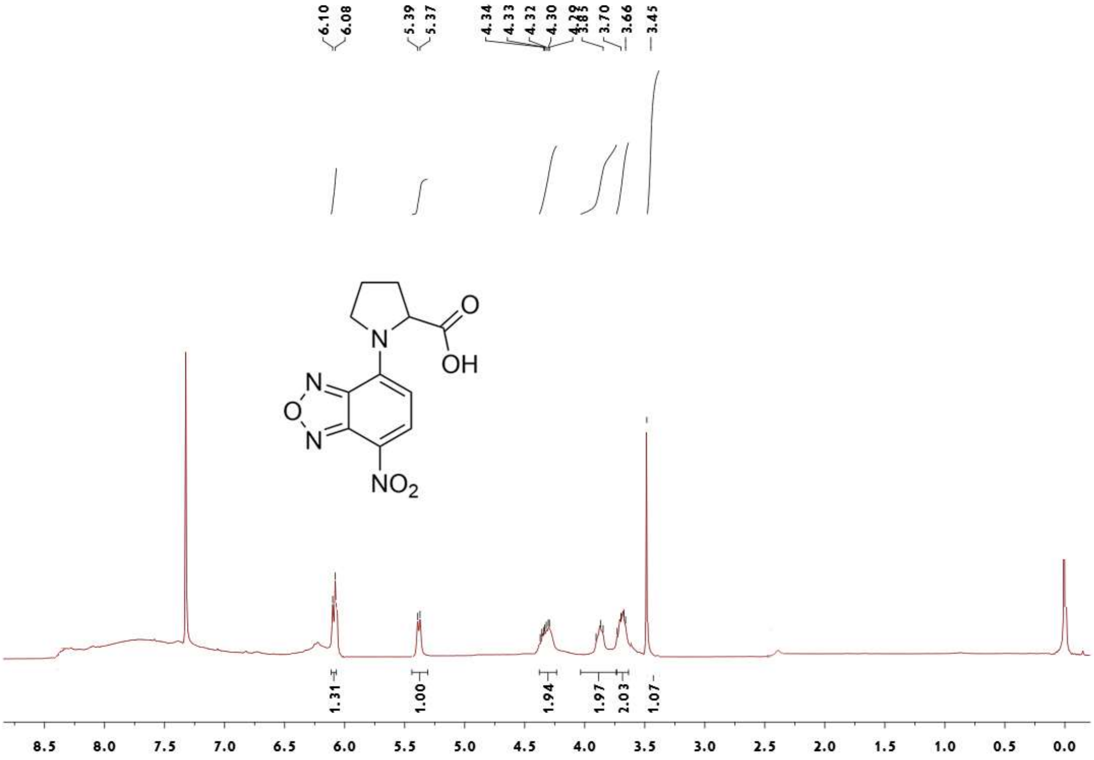

Figure S5. ^{1}H-NMR spectrum of compound 3

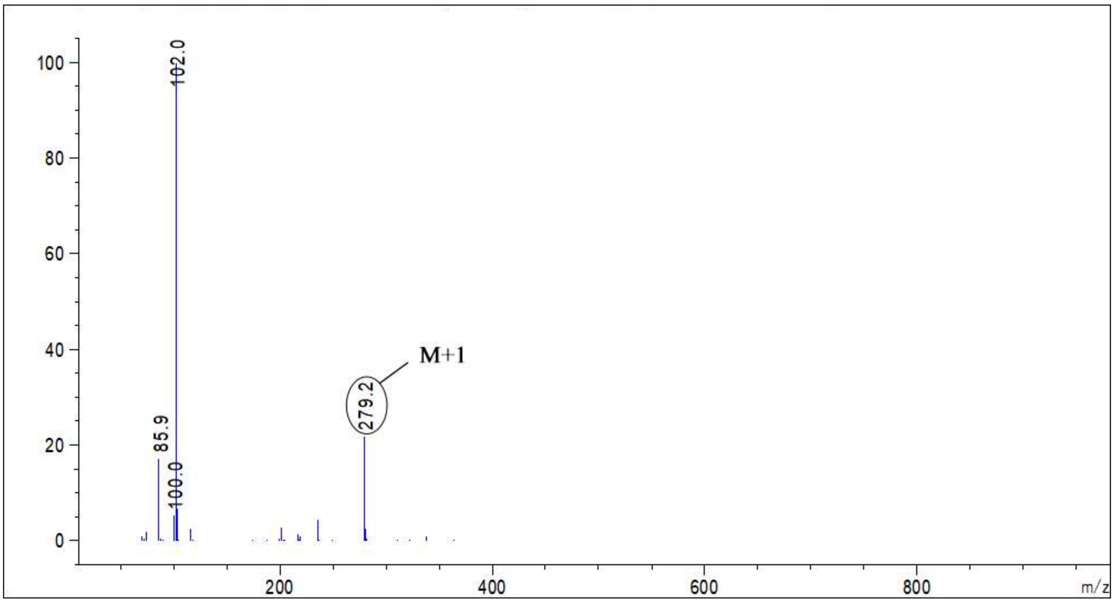

Figure S6. ESI-MS spectra of compound 3
